# Who declines to respond to the reactions to race module?: findings from the South Carolina Behavioral Risk Factor Surveillance System, 2016–2017

**DOI:** 10.1186/s12889-021-11748-y

**Published:** 2021-09-19

**Authors:** Aditi Srivastav, Kaitlynn Robinson-Ector, Colby Kipp, Melissa Strompolis, Kellee White

**Affiliations:** 1 Children’s Trust of South Carolina, Columbia, SC USA; 2 Department of Health Policy and Management, University of Maryland School of Public Health, College Park, MD USA; 3 Department of Psychology, University of South Carolina, Columbia, SC USA

**Keywords:** Racial discrimination, Reactions to race, Racism, Non-Response, Behavioral risk factor surveillance, Health surveys

## Abstract

**Background:**

The inclusion of self-reported differential treatment by race/ethnicity in population-based public health surveillance and monitoring systems may provide an opportunity to address long-standing health inequalities. While there is a growing trend towards decreasing response rates and selective non-response in health surveys, research examining the magnitude of non-response related to self-reported discrimination warrants greater attention. This study examined the distribution of sociodemographic variables among respondents and non-respondents to the South Carolina Behavioral Risk Factor Surveillance System (SC-BRFSS) Reactions to Race module (6-question optional module capturing reports of race-based treatment).

**Methods:**

Using data from SC-BRFSS (2016, 2017), we examined patterns of non-response to the Reactions to Race module and individual items in the module. Logistic regression models were employed to examine sociodemographic factors associated with non-response and weighted to account for complex sampling design.

**Results:**

Among 21,847 respondents, 15.3% were non-responders. Significant differences in RTRM non-response were observed by key sociodemographic variables (e.g., age, race/ethnicity, labor market participation, and health insurance status). Individuals who were younger, Hispanic, homemakers/students, unreported income, and uninsured were over-represented among non-respondents. In adjusted analyses, Hispanics and individuals with unreported income were more likely to be non-responders in RTRM and across item, while retirees were less likely to be non-responders. Heterogeneity in levels of non-responses were observed across RTRM questions, with the highest level of non-response for questions assessing differential treatment in work (54.8%) and healthcare settings (26.9%).

**Conclusions:**

Non-responders differed from responders according to some key sociodemographic variables, which could contribute to the underestimation of self-reported discrimination and race-related differential treatment and health outcomes. While we advocate for the use of population-based measures of self-reported racial discrimination to monitor and track state-level progress towards health equity, future efforts to estimate, assess, and address non-response variations by sociodemographic factors are warranted to improve understanding of lived experiences impacted by race-based differential treatment.

**Supplementary Information:**

The online version contains supplementary material available at 10.1186/s12889-021-11748-y.

## Introduction

There is an extensive scientific literature documenting the persistence of racial/ethnic health inequities [1, 2]. Research suggests that the persistence of racial/ethnic health inequities in part reflects unique psychosocial stressors, such as exposure to structural, cultural, personally-mediated, and internalized racism [2, 3]. A growing body of research shows that self-reported experiences of racial discrimination adversely impact health behaviors, pre-clinical indicators of disease, physical and mental health conditions, health care utilization and mortality [4–7]. While this work has greatly improved our understanding of health inequalities, the use of population-based measures of racial discrimination to monitor and track bias to inform effective public health practices and policies remains limited.

Population-based health surveys provide important information about the health and well-being of the U.S. population and serves as the foundation for national objectives. The Behavioral Risk Factor Surveillance System (BRFSS) was designed to collect state-level data on adults to assess health-related risk behaviors, chronic health conditions, and use of preventive services for public health programming, planning, monitoring, and evaluation [8]. Data are collected by telephone interviews of representative samples within each state and consists of a standardized core questionnaire (with fixed core, rotating core, and emerging core questions consistently collected), optional modules (questions on specific topics that states elect to use), and state-added questions (questions not part of the official BRFSS questionnaire). The survey is conducted daily, by landline and cell phone, to non-institutionalized adults who are eighteen years or older. Initially piloted in 2002, the Reactions to Race module (RTRM) is optional and consists of questions assessing socially-assigned race, race consciousness, perceptions of differential treatment by race/ethnicity in healthcare and work settings, and experiences of emotional and physical symptoms resulting from race-based treatment [9]. Individual questions and composite measures utilizing the RTRM have been associated with health care utilization, preventive screenings, health behaviors, and health outcomes [10–16]. It is one of the few measures of racial discrimination that is captured in a state-level population-based health survey that can track and monitor racial discrimination relative to population health and guide state and local public health practices and policies to address racial health inequalities.

Since the initiation of the BRFSS, researchers have been concerned about the impact of non-response bias on the interpretation and inferences drawn from survey data [17, 18]. Studies have shown that the median response rate for the BRFSS is 48% [19]. Reasons for non-response include the method of survey administration, length of the questionnaire, and reluctance to answer sensitive questions. Additionally, sub-groups of individuals (e.g., Blacks, Hispanics, Asians, and Native Americans, low income) are more likely to be underrepresented in population-based surveys [19]. Non-response may lead to reduced power and generalizability of studying findings if those who participate are systematically different from those who do not. While response rates for US population-based surveys, such as BRFSS continue to show a steady decline (median response rate: 2011–49.7%; 2017–45.9%), assessing nonresponse bias is ever more important [20]. There may be informative differences between responders and non-responders and understanding these differences may have important implications for monitoring and taking action to address racial health inequalities. However, less attention has been given to the magnitude of nonresponse associated with the BRFSS RTRM and little is known about characteristics of responders and non-responders.

Since 2002, a total of 23 states and the District of Columbia have administered the RTRM for at least 1 year. Even fewer states administered the RTRM in consecutive years. Most recently, the South Carolina BRFSS, employed the RTRM in consecutive years (2016 and 2017). We leverage this opportunity to compare the distribution of sociodemographic variables among respondents and non-respondents to the SC-BRFSS RTRM and RTRM items. We expect to find differences in the distribution of sociodemographic variables by response type. Findings related to item-specific non-response bias could potentially identify populations under-represented in capturing experiences of and reactions to discrimination reported by the SC-BRFSS.

## Methods

This study utilized data from the 2016 and 2017 South Carolina Behavioral Risk Factor Surveillance System (SC-BRFSS). The SC-BRFSS is managed by the South Carolina Department of Health and Environmental Control (SCDHEC). The survey was administered by the University of South Carolina’s Institute of Public Service and Policy Research (administration has subsequently been transferred to Winthrop University). To adjust for both sampling techniques and non-response, population weights assigned by the CDC were used (CDC, 2014). In 2016, the survey reached 11,236 respondents and in 2017, the survey reached 11,311 respondents. SC-BRFSS response rates in 2016 and 2017 were 45.8 and 47.3%, respectively, which is slightly higher than average national BRFSS response rates [21, 22]. The sample was further restricted to those who identified race/ethnicity as (Non-Hispanic) Black, Hispanic, or (Non-Hispanic) White, (with other racial/ethnic groups omitted due to small sample sizes), and who had complete interviews for the core survey and without missing data for key sociodemographic variables (i.e., education level, sex, race, age, and labor status participation), yielding a final analytic sample of 21,847 (2016: *n* = 10,886 respondents; 2017: *n =* 10,961 respondents).

The Children’s Trust of South Carolina, a nonprofit organization focused on preventing child abuse and neglect, researchers at the University of South Carolina School of Public Health, and SCDHEC partnered to finance the inclusion of the Reactions to Race Module (RTRM) in two consecutive years. RTRM, developed by the CDC Measures of Racism Work Group in 2001, is comprised of six-questions (see Table 1). Respondents are asked the RTRM questions after the core questions and towards the end of the survey. A dichotomous RTRM non-response variable was created based on respondents who were missing on all of the RTRM questions (“non-responders”) and individuals answering at least one RTRM question (“responders). RTRM item-specific non-response variable was assessed for each of the 6 RTRM questions, with individuals for whom responses were missing or who selected “refused,” considered non-responders, and those with a valid response or selected “don’t know/not sure” considered responders.

**Table 1:** Reactions to Race constructs included in the South Carolina Behavioral Risk Factor Surveillance System, 2016–2017

RTRM Construct	Survey Question	Response Categories
Socially-assigned race	How do other people usually classify you in this country? Would you say: White, Black or African American, Hispanic or Latino, Asian, Native Hawaiian or Other Pacific Islander, American Indian or Alaska Native, or some other group?	1: White 2: Black or African American 3: Hispanic or Latino 4: Asian 5: Native Hawaiian or Other Pacific Islander 6: American Indian or Alaska Native 7: Don’t know/Not sure 8: Some Other Group 9: Refused
Race consciousness	How often do you think about your race? Would you say never, once a year, once a month, once a week, once a day, once an hour, or constantly?	1: Never 2: Once a year 3: Once a month 4: Once a week 5: Once a day 6: Once an hour 7: Don’t know/Not sure 8: Constantly 9: Refused
Differential treatment	Within the past 12 months at work, do you feel you were treated worse than, the same as, or better than people of other races?	1: Worse than other races 2: The same as other races 3: Better than other races 4: Worse than some races, better than others 5: Only encountered people of the same race 7: Don’t know/Not sure 9: Refused
	Within the past 12 months, when seeking health care, do you feel your experiences were worse than, the same as, or better than for people of other races?	1: Worse than other races 2: The same as other races 3: Better than other races 4: Worse than some races, better than others 5: Only encountered people of the same race 6: No health care in past 12 months 7: Don’t know/Not sure 9: Refused
Reactions to differential treatment	Within the past 30 days, have you experienced any physical symptoms, for example, a headache, an upset stomach, tensing of your muscles, or a pounding heart, as a result of how you were treated based on your race?	1: Yes 2: No 7: Don’t know/Not Sure 9: Refused
	Within the past 30 days, have you felt emotionally upset, for example angry, sad, or frustrated, as a result of how you were treated based on your race?	

Sociodemographic characteristics of interest included sex, age, race/ethnicity, educational attainment, income, employment status, health insurance status, and geographic residence. Age was categorized into six groups: 18–34; 35–44; 45–64; and 65+. Educational attainment was categorized as: less than high school, high school graduate, some college, and college graduate. Income was measured as: < $15,000, $15,000 to $24,999, $25,000 to $34,999, $35,000 to $49,999, and $50,000 or more. Employment status was categorized as: employed/self-employed, not employed, retired, homemaker/student; and unable to work. Health insurance status was assessed as insured and uninsured. Geographic region of residence was classified as urban and rural.

### Statistical analysis

Comparing response rates across subgroups is one of the most common methods to assess non-response bias [19]. Employing similar analytic strategies as prior research [23], in 2020 estimates were generated using data from SC-BRFSS (2016, 2017) to examine non-responder distributions for the overall RTRM and the 6 RTRM questions and compared across sociodemographic characteristics. It is assumed that there is no evidence of nonresponse bias (so long as the subgroup variables are the only possible causes of response bias) if response rates are similar across subgroups; if rates are dissimilar, post-collection statistical adjustments can be made (e.g., weighting, multiple imputation) to reduce bias in population estimates resulting from nonresponse probabilities [24]. Differences between groups were examined using χ2 for all categorical variables. Logistic regression was used to determine whether factors were associated with response type adjusted for all sociodemographic variables. Sampling weights were applied to account for complex sampling design of the BRFSS. Data management and analyses were performed using SAS version 9.4 (SAS Institute Inc., Cary, NC). Institutional review board approval was not required for this study because the SC-BRFSS is a public use dataset and does not meet the criteria of human subjects research.

## Results

Our sample size was comprised of 21,847 adults in the 2016 and 2017 SC-BRFSS surveys. Survey results were calculated on values using CDC’s weighting procedures and are presented in percentages in Table 2. The study sample was 52.3% female, 25.4% Black, 5.0% Hispanic, and 66.5% White respondents. More respondents graduated from high school (30.0%) than from college (24.0%). A little over a third of the sample reported making $50,000 or more a year and over half of the sample reported being employed (55.4%). Among all those completing the SC-BRFSS, 84.7% agreed to complete the 6-question RTRM (respondents) and 156.3% declined to answer the RTRM questions (non-responders).

**Table 2:** Distribution of key sociodemographic characteristics by response to the Reactions to Race module, South Carolina Behavioral Risk Factor Surveillance System, 2016–2017

Characteristic		Weighted^1^ response to RTRM (%)		
	Total %	Responder^2^	Non-responder^3^	*p*-value^4^
	(*n* = 21,847)	(*n* = 19,380)	(*n* = 2467)	
Overall		84.7	15.3	
Sex				0.0758
Male	47.7	84.0	16.0	
Female	52.3	85.3	14.7	
Age				< 0.0001
18–34	29.4	77.9	22.1	
35–44	15.6	83.1	17.0	
45–64	33.2	87.6	12.4	
65+	21.8	90.5	9.5	
Race/ethnicity				0.0003
Black	25.4	83.8	16.2	
Hispanic	5.0	78.3	21.7	
White	66.5	85.5	14.5	
Education				0.8175
< HS	14.1	85.0	15.0	
HS graduate	30.0	84.2	15.8	
Some college	32.0	85.1	14.9	
College Graduate	24.0	84.5	15.5	
Income (per year)				< 0.0001
Less than $15,000	9.5	84.2	15.8	
$15,000–$24,999	15.9	85.5	14.5	
$25,000–$34,999	9.0	86.1	13.9	
$35,000–$49,999	12.5	87.1	12.9	
$50,000 or more	35.6	86.0	14.1	
Missing	17.4	79.0	21.0	
Employment status				< 0.0001
Employed or self employed	55.4	83.3	16.7	
Not employed	5.6	82.6	17.4	
Retired	19.3	90.7	9.3	
Homemaker / Student	10.0	81.6	18.4	
Unable to work	9.6	85.9	14.1	
Health insurance status				0.0002
Insured	86.8	85.3	14.7	
Uninsured	13.2	80.6	19.4	
Geographic residence				
Urban	72.3	89.8	10.3	0.1233
Rural	27.7	88.7	11.3	

*RTRM* Reactions To Race

^1^ Responses weighted according to guidelines provided by the Centers for Disease Control and Prevention

^2^ Responder = BRFSS survey participant who also answered the 6-question Reactions to Race module

^3^ Non-responder = BRFSS survey participant who declined to answer the 6-question Reactions to Race module

^4^*P*-values from the chi-square test of independence are reported for differences between respondent and non-respondents

When comparing responders to non-responders, there were significant differences by age, race/ethnicity, income, employment status, and health insurance status. Individuals below the age of 45 tended to be non-responders in comparison to older survey respondents. Hispanics had the highest level of non-response (21.7%) in comparison to Black (16.2%) and White (14.5%) respondents. Those respondents with missing income data (21%) had higher levels of non-response compared to those with reported income. Retired respondents had the lowest level of non-response (9.3%) while homemaker and student respondents have the highest level of non-response (18.4%). Individuals who were uninsured (19.4%) had a higher percentage of non-response in comparison to those who reported health insurance (14.7%).

RTRM item-non-response is presented by race/ethnicity (Table 3). RTRM item non-response levels varied, with the highest levels of non-response observed for the questions about differential treatment in work (54.8%) and healthcare (26.9%) settings. Non-response levels for questions about socially-assigned race, race-consciousness, and emotional and physical reactions to race-based treatment ranged between 16.3–18.8%. Overall, Hispanic non-responders differed significantly from Black and White non-responders across RTRM items, with the exception of differential treatment in healthcare settings. Hispanic respondents were significantly more likely to have higher non-response to the questions about socially-assigned race, race consciousness, and emotional and physical reactions to race-based treatment in comparison to Black and White respondents. For differential treatment in work settings, Hispanic respondents (47.6%) had the lowest non-response in comparison to Black (54.5%) and White respondents (55.4%).

**Table 3:** Weighted^1^ Reactions to Race Module item-nonresponse by race/ethnicity, South Carolina Behavioral Risk Factor Surveillance System, 2016–2017

	Total (*N =* 21,847)	Black (*N* = 5844)	Hispanic (*N* = 542)	White (*N* = 14,562)	*p*-value^3^
	Non-responder^2^ (%)	Non-responder (%)	Non-responder (%)	Non-responder (%)	
Socially-Assigned Race	16.3	17.2	25.2	15.2	<.0001
Race Consciousness	18.8	19.5	26.9	17.9	<.0001
Differential Treatment in Work Setting	54.8	54.5	47.6	55.4	0.0175
Differential Treatment in Healthcare Setting	26.9	25.9	29.2	27.0	0.4720
Emotional Reactions	16.4	17.3	23.7	15.4	<.0001
Physical Reactions	16.5	17.4	23.5	15.5	<.0001

*RTRM* Reactions To Race

^1^ Responses weighted according to guidelines provided by the Centers for Disease Control and Prevention

^2^ Non-responder = BRFSS survey participant who declined to answer a Reactions to Race Module item

^3^*P*-value from the chi-square test of independence are reported for differences by race/ethnicity by RTRM item non-response

The distribution of sociodemographic characteristics by RTRM item non-response is presented in Table 4. Across all of the RTRM items, individuals with younger age, unreported income, and uninsured were consistently overrepresented among non-responders. Differences in the distribution of sex by non-response were observed for select RTRM items. Females had higher levels of non-response to the socially-assigned race and reactions to differential treatment questions and men has higher levels of non-response to the differential treatment in work setting question.

**Table 4:** Distribution of sociodemographic characteristics by RTRM item non-response, South Carolina Behavioral Risk Factor Surveillance System, 2016–2017

	Socially-Assigned Race		Race consciousness		Experienced Differential Treatment				Reactions to Differential Treatment			
Characteristic					Work		Healthcare		Emotional		Physical	
	Non-responder (%)	*p*-value	Non-responder (%)	*p*-value	Non-responder (%)	*p*-value	Non-responder (%)	*p*-value	Non-responder (%)	*p*-value	Non-responder (%)	*p*-value
Sex												
Male	17.2	0.0374	19.5	0.0952	49.9	<.0001	27.4	0.2186	17.3	0.0268	17.3	0.0318
Female	15.6		18.1		59.2		26.4		15.5		15.7	
Age												
18–34	22.9	<.0001	24.8	<.0001	46.0	<.0001	28.2	<.0001	22.7	<.0001	22.8	<.0001
35–44	17.5		20.0		37.3		27.1		18.2		18.2	
45–64	13.5		15.7		48.5		23.7		13.4		13.6	
65+	11.0		14.4		88.6		29.7		11.0		11.0	
Education												
< HS	17.3	0.6009	18.8	0.9995	69.1	<.0001	31.0	0.0012	17.1	0.8051	17.3	0.6427
HS graduate	16.7		18.8		56.2		26.9		16.6		16.9	
Some college	15.8		18.7		54.0		25.7		15.9		15.9	
College graduate	16.1		18.8		45.5		25.8		16.3		15.9	
Income (per year)												
Less than $15,000	16.9	<.0001	18.1	<.0001	76.2	<.0001	29.1	<.0001	16.9	<.0001	17.2	<.0001
$15,000–$24,999	15.4		17.6		63.6		25.8		15.9		16.4	
$25,000–$34,999	15.4		17.4		53.9		24.2		14.4		14.3	
$35,000–$49,999	13.5		16.0		48.6		21.8		13.7		13.8	
$50,000 or more	14.5		17.0		40.9		23.0		14.8		14.7	
Missing	23.2		26.5		68.3		39.6		22.8		22.8	
Employment status												
Employed / self employed	17.5	< .0001	19.7	<.0001	21.6	*	24.3	<.0001	17.7	<.0001	17.6	<.0001
Not employed	18.2		20.1		63.5		29.6		18.9		18.1	
Retired	10.5		13.9		100.0		29.6		10.5		10.5	
Homemaker / Student	19.0		22.0		100.0		29.8		19.1		19.8	
Unable to work	16.0		17.5		100.0		29.5		15.0		16.2	
Health insurance status												
Insured	15.7	<.0001	18.1	0.0001	55.4	0.0042	25.9	<.0001	15.7	<.0001	15.8	<.0001
Uninsured	21.0		23.2		50.7		32.6		21.1		21.1	
Geographic residence												
Urban	11.3	0.1382	13.7	0.0909	50.3	<.0001	22.3	0.1930	11.3	0.0943	11.3	0.0767
Rural	12.3		15.0		57.3		23.4		12.5		12.6	

*RTRM* Reactions To Race;

^1^ Responses weighted according to guidelines provided by the Centers for Disease Control and Prevention;

* A *p*-value was not generated for this portion of the analysis

Table 5 shows the associations between sociodemographic characteristics and response type to the RTR module and RTRM items. When adjusting for sociodemographic characteristics, Hispanics respondents consistently had a higher odds of non-response in comparison to White respondents in the RTRM (OR = 1.36; 95% CI: 1.16, 1.61) and across all items, with the exception of differential treatment in healthcare. Respondents with unreported income had a higher odds of non-response in the RTRM (OR = 1.58; 95% CI: 1.20, 2.08). This association was generally robust across all RTRM items, with the exception of differential treatment at work (OR = 1.36; 95% CI: 0.97, 1.91). Retirees had a lower odds of non-response in the RTRM (OR = 0.75; 95% CI: 0.61, 0.92). This association was observed across all RTRM items, with the exception of differential treatment in health care, where retirees had a higher odds of non-response (OR = 1.35; 95% CI: 1.18, 1.55) in comparison to employed respondents.

**Table 5:** Sociodemographic factors associated with response type in South Carolina Behavioral Risk Factor Surveillance System, 2016–2017

	RTRM	RTRM Items					
		Socially-Assigned Race	Race Consciousness	Differential Treatment at Work	Differential treatment in Healthcare	Emotional Reactions	Physical Reactions
Variable	OR_adj_^a^ (95% CI)	OR_adj_^a^ (95% CI)	OR_adj_^a^ (95% CI)	OR_adj_^b^ (95% CI)	OR_adj_^a^ (95% CI)	OR_adj_^a^ (95% CI)	OR_adj_^a^ (95% CI)
Sex							
Male	1.00	1.00	1.00	1.00	1.00	1.00	1.00
Female	0.87 (0.76, 1.01)	0.86 (0.75, 0.98)	0.91 (0.80, 1.03)	0.83 (0.71, 0.97)	0.90 (0.82, 1.00)	0.85 (0.74, 0.98)	0.84 (0.73, 0.97)
Age							
18–34	1.00	1.00	1.00	1.00	1.00	1.00	1.00
35–44	1.61 (1.25, 2.06)	1.39 (1.10, 1.76)	1.16 (0.93, 1.44)	0.72 (0.56, 0.93)	0.72 (0.60, 0.86)	1.39 (1.10, 1.76)	1.37 (1.09, 1.74)
45–64	1.53 (1.20 1.96)	1.32 (1.04, 1.67)	1.18 (0.95, 1.46)	0.86 (0.67, 1.09)	0.99 (0.82, 1.18)	1.45 (1.14, 1.83)	1.40 (1.11, 1.77)
65+	1.13 (0.93, 1.37)	1.03 (0.86, 1.23)	0.96 (0.82, 1.12)	0.77 (0.63, 0.95)	0.83 (0.73, 0.94)	1.07 (0.89, 1.28)	1.04 (0.87, 1.25)
Race/ethnicity							
White	1.00	1.00	1.00	1.00	1.00	1.00	1.00
Black	0.94 (0.63, 1.39)	1.22 (0.85, 1.74)	1.21 (0.87, 1.69)	0.96 (0.67, 1.37)	0.78 (0.57, 1.07)	1.03 (0.70, 1.52)	0.99 (0.68, 1.45)
Hispanic	1.36 (1.16, 1.61)	1.36 (1.17, 1.60)	1.31 (1.13, 1.50)	1.25 (1.05, 1.50)	1.04 (0.92, 1.16)	1.35 (1.16, 1.57)	1.33 (1.14, 1.55)
Education							
< HS	1.00	1.00	1.00	1.00	1.00	1.00	1.00
HS graduate	1.03 (0.82, 1.30)	0.95 (0.76, 1.18)	1.00 (0.81, 1.23)	0.96 (0.73, 1.27)	0.91 (0.78, 1.07)	0.94 (0.76, 1.18)	0.94 (0.76, 1.17)
Some college	0.94 (0.73, 1.20)	0.88 (0.69, 1.11)	1.01 (0.81, 1.26)	0.92 (0.68, 1.23)	0.88 (0.74, 1.05)	0.89 (0.71, 1.13)	0.89 (0.70, 1.12)
College graduate	0.81 (0.62, 1.06)	0.75 (0.59, 0.97)	0.89 (0.70, 1.12)	0.82 (0.61, 1.11)	0.82 (0.68, 0.98)	0.76 (0.59, 0.98)	0.73 (0.57, 0.95)
Income (per year)							
Less than $15,000	1.00	1.00	1.00	1.00	1.00	1.00	1.00
$15,000–$24,999	1.06 (0.80, 1.40)	1.03 (0.79, 1.50)	1.12 (0.88, 1.43)	1.17 (0.84, 1.62)	0.97 (0.80, 1.18)	1.08 (0.83, 1.40)	1.12 (0.86, 1.45)
$25,000–$34,999	0.86 (0.61, 1.19)	0.94 (0.69, 1.29)	0.99 (0.74, 1.34)	0.96 (0.66, 1.40)	0.84 (0.67, 1.05)	0.81 (0.59, 1.11)	0.80 (0.58, 1.10)
$35,000–$49,999	0.76 (0.55, 1.04)	0.76 (0.56, 1.03)	0.87 (0.65, 1.15)	0.84 (0.58, 1.21)	0.73 (0.58, 0.91)	0.75 (0.55, 1.02)	0.75 (0.55, 1.02)
$50,000 or more	0.78 (0.58, 1.03)	0.77 (0.57, 1.02)	0.88 (0.69, 1.13)	0.76 (0.54, 1.05)	0.78 (0.63, 0.95)	0.75 (0.57, 0.99)	0.74 (0.56, 0.97)
Missing	1.58 (1.20, 2.08)	1.68 (1.30, 2.19)	1.79 (1.40, 2.28)	1.36 (0.97, 1.91)	1.77 (1.46, 2.15)	1.59 (1.22, 2.08)	1.57 (1.21, 2.02)
Employment status							
Employed / self employed	1.00	1.00	1.00		1.00	1.00	1.00
Not employed	1.08 (0.76, 1.41)	1.01 (0.75, 1.37)	1.02 (0.76, 1.37)		1.31 (1.04, 1.67)	1.06 (0.79, 1.43)	0.99 (0.73, 1.35)
Retired	0.75 (0.61, 0.92)	0.74 (0.61, 0.90)	0.83 (0.70, 0.98)		1.35 (1.18, 1.55)	0.74 (0.61, 0.90)	0.73 (0.60, 0.88)
Homemaker / Student	0.89 (0.67, 1.20)	0.86 (0.65, 1.15)	0.95 (0.74, 1.22)		1.22 (0.99, 1.49)	0.89 (0.67, 1.18)	0.91 (0.69, 1.20)
Unable to work	1.09 (0.85, 1.38)	1.12 (0.89, 1.41)	1.10 (0.88, 1.36)		1.37 (1.16, 1.63)	1.00 (0.79, 1.26)	1.10 (0.88, 1.38)
Health insurance status							
Insured	1.00	1.00	1.00	1.00	1.00	1.00	1.00
Uninsured	1.11 (0.83, 1.23)	1.00 (0.83, 1.20)	0.97 (0.85, 1.10)	1.04 (0.86, 1.25)	0.78 (0.68, 0.90)	1.02 (0.79, 1.26)	1.04 (0.86, 1.25)
Geographic residence							
Urban	1.00	1.00	1.00	1.00	1.00	1.00	1.00
Rural	0.98 (0.85, 1.13)	1.00 (0.87, 1.15)	1.23 (1.09, 1.38)	0.85 (0.73, 1.00)	1.03 (0.94, 1.15)	0.98 (0.85, 1.12)	0.97 (0.84, 1.12)

*RTRM* Reactions To Race;

^a^Models adjusted for sex, age, race/ethnicity, education, income, employment status, health insurance status, and geographic residence

^b^Models adjusted for sex, age, race/ethnicity, education, income, health insurance status, and geographic residence

In sensitivity analyses, we explored the distribution of sociodemographic factors by RTRM item non-response for each racial/ethnic group (Supplementary Tables 1, 2 and 3). Overall, age, income, and insurance status were most consistently related to RTRM item non-response among Black and White respondents. Additionally among White respondents, employment status, was also consistently related to levels of non-response. Notably among Hispanics respondents, fewer sociodemographic characteristics were consistently related to RTRM item specific non-response.

## Discussion

To our knowledge, this is the first study to examine sociodemographic characteristics associated with RTRM non-response using a BRFSS sample. Overall, 15.3% of survey respondents did not respond to the RTRM. Patterns of non-response varied across population subgroups. We observed heterogeneity in non-response across RTRM items where differential treatment in work (54.8%) and healthcare settings (26.9%) had the highest levels of non-response. There were significant differences in non-response by age, race/ethnicity, socioeconomic status and employment status.

In this SC-BRFSS sample, response patterns to the RTRM varied across population subgroups. Individuals who identified as Hispanic, indicated unreported income, and were uninsured had higher percentages of non-response to the RTRM, while individuals who were older and retired had lower percentages of non-response. These categories are similar to the groups that are typically non-responders to the BRFSS core. BRFSS makes efforts to weight the sample data by age, gender and race/ethnicity, with more recent administrations of the BRFSS employing a raking weighting methodology that additionally considers marital status and socioeconomic status (e.g., educational attainment and property owner/rental status) to reduce bias and improve representativeness not only in the BRFSS core but potentially in optional modules.

RTRM item-specific non-response ranged from 16.3 to 54.8%, with the highest levels of non-response observed among the questions inquiring about differential treatment in work and healthcare settings. Reasons for the high level of non-response for differential treatment in work settings may be attributed to labor market participation. In our sample, only 55.4% of the population reported being currently employed. The other portion of respondents comprised of those who reported being unemployed, retired, homemaker/student, or unable to work, and may have decided to not answer the question because of their work status. The question querying respondents about differential treatment in healthcare settings also had relatively increased levels of non-response (overall, 26.9%). We do not suspect this is a function of health insurance status since many respondents reported having some type of health insurance. However, research assessing specific psychometric properties of these questions may be needed to further refine this important question about one’s lived experience.

Racial/ethnic differences were observed in item-specific non-response. In general, Black and White respondents had relatively similar levels of non-response across RTRM items. Hispanic respondents had the lowest levels of non-response for the questions about differential treatment in work settings. Labor market participation and age of Hispanic respondents may partially explain these differences. Moreover, Hispanic respondents had the highest levels of non-response to the questions about socially-assigned race, race-consciousness, and emotional and physical reactions to race-based treatment. Willingness and motivation to respond to the aforementioned questions may be dictated by several factors. Hispanics respondents may experience a unique constellation of structural disadvantages (e.g., immigration legal status and language proficiency) that intersect with the traditional axis of inequality that emphasizes race which may influence actual and perceived encounters with negative and differential treatment [25]. A higher non-response to these questions may also be a function of the classification of Hispanics/Latinos as an ethnicity rather than a racial group. Prior research documents that directives established by the Office of Management and Budget (OMB), which mandates the standards and provides guidance for the collection of race and ethnicity data in the US, may not provide relevant options for describing Hispanic racial identity and the options can be confusing because it may not cohere with Hispanics’ understanding of race [26, 27]. The extent to which differential responses to these questions and for people of different racial background, ethnic identity, or nativity, which are all important correlates of one’s racialized lived experience, is not clear and needs to be further explored. It is possible that RTRM items carry sociopolitical implications and can evoke associations, feelings, or different judgments in the minds of respondents that give rise to particular interpretations or function differently by race/ethnicity [28]. Notably, Hispanics only represented 5% of respondents, thus, the observed levels of non-response need to be interpreted with caution. Future studies should assess differential item functioning of the RTRM items by race/ethnicity with sufficient sample sizes.

To our knowledge, BRFSS does not report response rates of optional modules in the annual data quality report. We are aware of one prior study that assessed non-response to a BRFSS optional module [23]. Crouch et al. examined the SC-BRFSS Adverse Childhood Exposures (ACE) optional module and documented differences between responders and non-responders by sex, age, race/ethnicity, education, income, and rurality in bivariate analyses. Overall, there were some similarities in the range of sociodemographic factors (e.g., age, race/ethnicity, and income) associated with non-response as evidenced from our bivariate analyses. However, unlike Crouch et al. (2018), we did not observe differences in response type by sex, educational attainment or geographic region of residence for the overall RTRM. Yet, for few RTRM items, sex and educational attainment were related to non-response. For example, differential treatment in work settings, females were over-represented among non-respondents. Further, we identified retirees and uninsured respondents overrepresented among overall RTRM non-responders. Moreover, in adjusted analyses, Hispanic respondents, those with unreported income, and retirees were most consistently associated with non-response in the RTRM and across RTRM items. This suggest that when studying race-based differential treatment and health, associations may be weaker among these groups and are likely to be conservative. It is not well known how non-response to BRFSS optional modules are affected by sociodemographic factors. Future studies estimating and assessing factors influencing RTRM and other optional module response levels may help to improve understanding of the extent of bias and its implication for interpreting results.

It is imperative that studies using these data consider employing analytic techniques that can accommodate patterns of missingness during the analysis stage. Typical approaches to compensate for variations in probability of selection and overall nonresponse, including weighting the sample data, may not fully address item-specific nonresponse. Prior research has shown that developing weights for a subset of respondents answering specific modules, for example, is not practical [29]. For data missing at random, considering approaches such as multiple imputation [17, 29, 30], full information maximum likelihood estimation [17, 30], and inverse probability weighting [31, 32] during the analysis stage are potential strategies to reduce bias and improve validity of the estimates. Further, RTRM nonresponse may also be a function of order effects. In South Carolina, the optional module is administered after core component questions and are often placed towards the end of the questionnaire.

As with any study using the BRFSS, we acknowledge that the data is limited by its retrospective, self-reported nature. Similar to questions about sensitive and stigmatizing life circumstances (e.g., abuse/neglect,) or health conditions (e.g., mental health, substance use), responding to experiences of race-based differential treatment may be subjected to recall and social desirability bias. While these results may be comparable to other states in the American South, they are not generalizable nationally given the limited sample size of Asians, Native Americans, and Hawaiian/Pacific Islanders. The RTRM was developed specifically for public health surveillance of racialized lived experiences in a US context. Despite these limitations, our paper has some important implications. Valid and reliable measures of experiences of racial discrimination in population-based surveys may be critical for guiding anti-racist action in public health practice and programs and informing anti-racist, data-driven policy implementation. This public health surveillance data is valuable for illuminating sources of inequities between specific populations. Additional research is needed to consider whether the observed factors influencing non-response in RTRM are consistent across other states with different proportions of Hispanics, Asians, and Native Americans, and multiracial populations groups. However, to provide this type of insight, it is incumbent among more states to administer this optional module as part of the BRFSS to provide additional insight regarding the contribution of racial discrimination to health and well-being.

## Conclusions

We observed that responders and non-responders to the RTRM and items varied across key sociodemographic variables. Examining patterns of differential non-response could provide insight towards our understanding of estimates and trends of self-reported discrimination and race-related differential treatment and associations with health outcomes collected in public health population-based monitoring and surveillance systems. Public health surveys such as the BRFSS are essential tools for identifying and monitoring population health inequalities. However, tools for collecting, monitoring, and tracking racialized lived experiences at the state and local levels are limited. Enhancing the utility and generalizability of the RTRM across diverse groups has the potential to serve as a state-wide population-based tool to understand the relationship between population health and self-reported racial discrimination in different settings, inform data-driven population-based practices and policies for improving health promotion, disease prevention and management, and evaluate progress towards achieving health equity through avoidable inequalities, historical, and contemporary injustices [28].

## Supplementary Information

**Additional file 1: Supplementary Table 1.** Distribution of sociodemographic characteristics by RTRM item non-response among Blacks respondents, South Carolina Behavioral Risk Factor Surveillance System, 2016–2017. **Supplementary Table 2.** Distribution of sociodemographic characteristics by RTRM item non-response among White respondents, South Carolina Behavioral Risk Factor Surveillance System, 2016–2017. **Supplementary Table 3.** Distribution of sociodemographic characteristics by RTRM item non-response among Hispanics respondents, South Carolina Behavioral Risk Factor Surveillance System, 2016–2017.

## Data Availability

The SC-BRFSS is a publicly available dataset (https://www.cdc.gov/brfss/data_documentation/index.htm). The datasets used and analyzed in the current study are available from the corresponding author on reasonable request.

## Abbreviations

BRFSS: Behavioral Risk Factor Surveillance System
CDC: Centers for Disease Control and Prevention
SCDHEC: South Carolina Department of Health and Environmental Control
SC-BRFSS: South Carolina Behavioral Risk Factor Surveillance System
RTRM: Reactions to Race module
