# Supplementary material for: Who declines to respond to the reactions to race module?: findings from the South Carolina Behavioral Risk Factor Surveillance System, 2016–2017
Source: BMC Public Health. 2021 Sep 19;21:1703. doi: 10.1186/s12889-021-11748-y (PMC8449882; doi:10.1186/s12889-021-11748-y)
Supplement: Supplementary file 1 — Additional file 1: Supplementary Table 1. Distribution of sociodemographic characteristics by RTRM item non-response among Blacks respondents, South Carolina Behavioral Risk Factor Surveillance System, 2016–2017. Supplementary Table 2. Distribution of sociodemographic characteristics by RTRM item non-response among White respondents, South Carolina Behavioral Risk Factor Surveillance System, 2016–2017. Supplementary Table 3. Distribution of sociodemographic characteristics by RTRM item non-response among Hispanics respondents, South Carolina Behavioral Risk Factor Surveillance System, 2016–2017. [file 12889_2021_11748_MOESM1_ESM.docx]

**Title:** Who Declines To Respond to the Reactions to Race Module?: Findings from the South Carolina Behavioral Risk Factor Surveillance System, 2016-2017

**Author:** Aditi Srivastav,^1^ Kaitlynn Robinson-Ector,^2^ Colby Kipp,^3^  Melissa Strompolis, ^1^ Kellee White^2^

**Author**

**Affiliations:** ^1^Children’s Trust of South Carolina

Columbia, South Carolina

^2^University of Maryland College Park School of Public Health

Department of Health Policy and Management

College Park, Maryland

^3^University of South Carolina – Department of Psychology

Columbia, South Carolina

**Corresponding**

**Author:** Kellee White, PhD, MPH

University of Maryland College Park School of Public Health

Department of Health Policy and Management

4200 Valley Drive, SPH BLDG 255, Suite 3310

College Park, Maryland 20742-2611

301 405 6125

[kwhite20@umd.edu](mailto:kwhite20@umd.edu)

**Appendix Materials**

1. Supplementary Table 1: Sociodemographic characteristics associated with Reactions to Race Module item non-response among Black Respondents, South Carolina Behavioral Risk Factor Surveillance System, 2016-2017
2. Supplementary Table 2: Sociodemographic characteristics associated with Reactions to Race Module item non-response among White Respondents, South Carolina Behavioral Risk Factor Surveillance System, 2016-2017
3. Supplementary Table 3: Sociodemographic characteristics associated with Reactions to Race Module item non-response among Hispanic Respondents, South Carolina Behavioral Risk Factor Surveillance System, 2016-2017

Supplementary Table 1: Distribution of sociodemographic characteristics by RTRM item non-response among Blacks respondents, South Carolina Behavioral Risk Factor Surveillance System, 2016-2017

|  | Socially-Assigned Race | | Race consciousness | | Experienced Differential Treatment | | | | Reactions to Differential Treatment | | | |
| --- | --- | --- | --- | --- | --- | --- | --- | --- | --- | --- | --- | --- |
| Characteristic |  | |  | | Work | | Healthcare | | Emotional | | Physical | |
|  | Non-responder  (%) | *p*-value | Non-responder  (%) | *p*-value | Non-responder  (%) | *p*-value | Non-responder  (%) | *p*-value | Non-responder  (%) | *p*-value | Non-responder  (%) | *p*-value |
| Sex |  | 0.4558 |  | 0.4743 |  | 0.7485 |  | 0.2679 |  | 0.2570 |  | 0.1646 |
| Male | 17.9 |  | 20.1 |  | 54.2 |  | 27.0 |  | 18.3 |  | 18.6 |  |
| Female | 16.7 |  | 19.0 |  | 54.8 |  | 25.1 |  | 16.5 |  | 16.4 |  |
| Age |  | <.0001 |  | 0.0022 |  | <.0001 |  | 0.3707 |  | <.0001 |  | <.0001 |
| 18 - 34 | 21.5 |  | 23.3 |  | 45.1 |  | 26.3 |  | 22.2 |  | 22.0 |  |
| 35 - 44 | 17.1 |  | 19.1 |  | 39.3 |  | 25.1 |  | 17.5 |  | 17.5 |  |
| 45 - 64 | 15.0 |  | 17.5 |  | 54.5 |  | 24.6 |  | 14.8 |  | 15.2 |  |
| 65+ | 13.2 |  | 16.1 |  | 89.5 |  | 28.9 |  | 12.1 |  | 12.5 |  |
| Education |  | 0.2385 |  | 0.1896 |  | <.0001 |  | 0.0007 |  | 0.1870 |  | 0.1562 |
| < HS | 16.5 |  | 18.1 |  | 70.1 |  | 30.4 |  | 16.7 |  | 17.1 |  |
| HS graduate | 18.7 |  | 21.3 |  | 56.6 |  | 28.0 |  | 18.9 |  | 18.9 |  |
| Some college | 17.3 |  | 19.5 |  | 49.0 |  | 23.6 |  | 17.3 |  | 17.7 |  |
| College graduate | 14.0 |  | 16.4 |  | 40.8 |  | 19.9 |  | 13.7 |  | 13.5 |  |
| Income (per year) |  | 0.0023 |  | 0.0007 |  | <.0001 |  | <.0001 |  | 0.0073 |  | 0.0036 |
| Less than $15,000 | 15.4 |  | 17.2 |  | 71.2 |  | 25.6 |  | 16.0 |  | 16.2 |  |
| $15,000-$24,999 | 16.6 |  | 18.4 |  | 55.7 |  | 25.0 |  | 17.5 |  | 17.8 |  |
| $25,000-$34,999 | 15.3 |  | 16.5 |  | 46.3 |  | 20.6 |  | 14.7 |  | 14.7 |  |
| $35,000-$49,999 | 16.0 |  | 18.9 |  | 42.4 |  | 21.0 |  | 16.1 |  | 16.3 |  |
| $50,000 or more | 14.6 |  | 17.2 |  | 39.1 |  | 19.9 |  | 14.3 |  | 14.0 |  |
| Missing | 24.2 |  | 27.3 |  | 66.5 |  | 39.9 |  | 23.5 |  | 23.9 |  |
| Employment status |  | 0.1326 |  | 0.1852 |  | * |  | 0.1494 |  | 0.0307 |  | 0.0584 |
| Employed / self employed | 18.3 |  | 20.7 |  | 23.5 |  | 23.6 |  | 18.5 |  | 18.6 |  |
| Not employed | 17.6 |  | 17.9 |  | 61.0 |  | 28.3 |  | 20.0 |  | 17.8 |  |
| Retired | 12.7 |  | 16.6 |  | 100.0 |  | 27.8 |  | 11.9 |  | 11.9 |  |
| Homemaker / Student | 18.2 |  | 21.2 |  | 100.0 |  | 28.3 |  | 18.2 |  | 19.4 |  |
| Unable to work | 15.5 |  | 16.3 |  | 100.0 |  | 28.5 |  | 15.0 |  | 16.3 |  |
| Health insurance status |  | 0.005 |  | 0.0055 |  | 0.5534 |  | <.0001 |  | 0.0015 |  | 0.0113 |
| Insured | 16.2 |  | 18.4 |  | 54.8 |  | 24.1 |  | 16.1 |  | 16.5 |  |
| Uninsured | 22.7 |  | 25.1 |  | 53.1 |  | 34.8 |  | 23.5 |  | 22.4 |  |
| Geographic residence |  | 0.5065 |  | 0.2362 |  | <.0001 |  | 0.3393 |  | 0.4577 |  | 0.3156 |
| Urban | 14.7 |  | 16.7 |  | 58.4 |  | 25.0 |  | 14.7 |  | 16.2 |  |
| Rural | 15.7 |  | 18.5 |  | 50.3 |  | 23.4 |  | 15.8 |  | 14.7 |  |

RTRM, Reactions To Race

^1^ Responses weighted according to guidelines provided by the Centers for Disease Control and Prevention

* A p-value was not generated for this portion of the analysis; Supplementary Table 2: Distribution of sociodemographic characteristics by RTRM item non-response White respondents, South Carolina Behavioral Risk Factor Surveillance System, 2016-2017

|  | Socially-Assigned Race | | Race consciousness | | Experienced Differential Treatment | | | | Reactions to Differential Treatment | | | |
| --- | --- | --- | --- | --- | --- | --- | --- | --- | --- | --- | --- | --- |
| Characteristic |  | |  | | Work | | Healthcare | | Emotional | | Physical | |
|  | Non-responder  (%) | *p*-value | Non-responder  (%) | *p*-value | Non-responder  (%) | *p*-value | Non-responder  (%) | *p*-value | Non-responder  (%) | *p*-value | Non-responder  (%) | *p*-value |
| Sex |  | 0.0314 |  | 0.0513 |  | <.0001 |  | 0.4596 |  | 0.0452 |  | 0.0562 |
| Male | 16.2 |  | 18.8 |  | 49.3 |  | 27.4 |  | 16.4 |  | 16.4 |  |
| Female | 14.2 |  | 17.0 |  | 61.1 |  | 26.6 |  | 14.5 |  | 14.6 |  |
| Age |  | <.0001 |  | <.0001 |  | <.0001 |  | <.0001 |  | <.0001 |  | <.0001 |
| 18 - 34 | 23.0 |  | 25.4 |  | 45.9 |  | 29.2 |  | 22.9 |  | 22.8 |  |
| 35 - 44 | 16.8 |  | 18.8 |  | 35.7 |  | 26.9 |  | 17.3 |  | 17.7 |  |
| 45 - 64 | 12.4 |  | 14.9 |  | 45.9 |  | 23.2 |  | 12.7 |  | 12.8 |  |
| 65+ | 10.3 |  | 14.0 |  | 88.4 |  | 29.9 |  | 10.6 |  | 10.6 |  |
| Education |  | 0.8766 |  | 0.5987 |  | <.0001 |  | 0.1004 |  | 0.8739 |  | 0.9087 |
| < HS | 15.5 |  | 16.7 |  | 72.9 |  | 30.8 |  | 15.3 |  | 16.4 |  |
| HS graduate | 15.2 |  | 17.2 |  | 56.9 |  | 26.8 |  | 15.2 |  | 15.4 |  |
| Some college | 14.7 |  | 18.2 |  | 56.1 |  | 26.3 |  | 15.1 |  | 15.0 |  |
| College graduate | 15.7 |  | 18.6 |  | 46.5 |  | 26.6 |  | 16.0 |  | 15.8 |  |
| Income (per year) |  | <.0001 |  | <.0001 |  | <.0001 |  | <.0001 |  | <.0001 |  | <.0001 |
| Less than $15,000 | 16.0 |  | 16.9 |  | 82.0 |  | 31.2 |  | 15.5 |  | 21.7 |  |
| $15,000-$24,999 | 13.6 |  | 16.2 |  | 70.8 |  | 26.1 |  | 13.9 |  | 15.9 |  |
| $25,000-$34,999 | 14.7 |  | 17.2 |  | 59.6 |  | 25.4 |  | 13.6 |  | 14.8 |  |
| $35,000-$49,999 | 12.1 |  | 14.6 |  | 51.6 |  | 21.8 |  | 12.5 |  | 13.5 |  |
| $50,000 or more | 14.2 |  | 16.9 |  | 41.4 |  | 23.6 |  | 14.7 |  | 12.4 |  |
| Missing | 21.5 |  | 25.2 |  | 28.8 |  | 39.8 |  | 21.8 |  | 14.6 |  |
| Employment status |  | <.0001 |  | <.0001 |  | * |  | <.0001 |  | <.0001 |  | <.0001 |
| Employed / self employed | 16.5 |  | 18.9 |  | 20.3 |  | 24.1 |  | 16.8 |  | 16.8 |  |
| Not employed | 16.8 |  | 20.3 |  | 64.6 |  | 28.7 |  | 16.7 |  | 16.7 |  |
| Retired | 9.7 |  | 13.1 |  | 100 |  | 30.2 |  | 10.0 |  | 10.0 |  |
| Homemaker / Student | 18.2 |  | 20.4 |  | 100 |  | 30.4 |  | 18.6 |  | 18.4 |  |
| Unable to work | 15.9 |  | 18.6 |  | 100 |  | 30.1 |  | 14.9 |  | 16.2 |  |
| Health insurance status |  | 0.0569 |  | 0.1391 |  | 0.0132 |  | 0.0277 |  | 0.0736 |  | 0.0246 |
| Insured | 14.9 |  | 17.6 |  | 55.9 |  | 26.5 |  | 15.1 |  | 15.1 |  |
| Uninsured | 18.2 |  | 20.3 |  | 50.4 |  | 31.0 |  | 18.2 |  | 19.0 |  |
| Geographic residence |  | 0.5575 |  | 0.7083 |  | <.0001 |  | 0.60 |  | 0.3949 |  | 0.4136 |
| Urban | 9.7 |  | 12.5 |  | 51.1 |  | 22.1 |  | 9.9 |  | 10 |  |
| Rural | 10.2 |  | 12.8 |  | 57.6 |  | 22.6 |  | 10.6 |  | 10.6 |  |

RTRM, Reactions To Race

^1^ Responses weighted according to guidelines provided by the Centers for Disease Control and Prevention

* A p-value was not generated for this portion of the analysis;

Supplementary Table 3: Distribution of sociodemographic characteristics by RTRM item non-response among Hispanics respondents, South Carolina Behavioral Risk Factor Surveillance System, 2016-2017

|  | Socially-Assigned Race | | Race consciousness | | Experienced Differential Treatment | | | | Reactions to Differential Treatment | | | |
| --- | --- | --- | --- | --- | --- | --- | --- | --- | --- | --- | --- | --- |
| Characteristic |  | |  | | Work | | Healthcare | | Emotional | | Physical | |
|  | Non-responder  (%) | *p*-value | Non-responder  (%) | *p*-value | Non-responder  (%) | *p*-value | Non-responder  (%) | *p*-value | Non-responder  (%) | *p*-value | Non-responder  (%) | *p*-value |
| Sex |  | 0.4126 |  | 0.1279 |  | 0.0001 |  | 0.4073 |  | 0.4908 |  | 0.2483 |
| Male | 23.3 |  | 23.3 |  | 37.3 |  | 27.2 |  | 22.1 |  | 21.0 |  |
| Female | 27.4 |  | 31.0 |  | 59.4 |  | 31.5 |  | 25.5 |  | 26.4 |  |
| Age |  | 0.3853 |  | 0.0625 |  | 0.0411 |  | 0.4571 |  | 0.1742 |  | 0.5015 |
| 18 - 34 | 27.4 |  | 26.7 |  | 48.1 |  | 27.6 |  | 23.5 |  | 24.8 |  |
| 35 - 44 | 25.8 |  | 34.4 |  | 41.2 |  | 35.2 |  | 29.8 |  | 25.0 |  |
| 45 - 64 | 19.3 |  | 19.6 |  | 48.9 |  | 26.7 |  | 17.9 |  | 19.3 |  |
| 65+ | 15.3 |  | 12.1 |  | 82.7 |  | 25.8 |  | 11.4 |  | 11.4 |  |
| Education |  | 0.5142 |  | 0.6658 |  | 0.7466 |  | 0.503 |  | 0.5522 |  | 0.6472 |
| < HS | 26.4 |  | 29.9 |  | 50.8 |  | 32.6 |  | 26.0 |  | 24.2 |  |
| HS graduate | 19.8 |  | 22.9 |  | 46.5 |  | 23.8 |  | 18.4 |  | 19.3 |  |
| Some college | 28.4 |  | 28.2 |  | 46.7 |  | 29.2 |  | 25.2 |  | 27.2 |  |
| College graduate | 27.9 |  | 25.3 |  | 41.8 |  | 31.4 |  | 25.6 |  | 24.7 |  |
| Income (per year) |  | 0.2186 |  | 0.1562 |  | 0.0088 |  | 0.2908 |  | 0.2466 |  | 0.2979 |
| Less than $15,000 | 37.5 |  | 37.2 |  | 66.0 |  | 39.2 |  | 36.4 |  | 37.2 |  |
| $15,000-$24,999 | 21.0 |  | 25.3 |  | 51.6 |  | 27.1 |  | 23.1 |  | 23.1 |  |
| $25,000-$34,999 | 27.3 |  | 29.4 |  | 44.0 |  | 29.9 |  | 25.1 |  | 25.1 |  |
| $35,000-$49,999 | 12.8 |  | 9.1 |  | 15.4 |  | 16.2 |  | 9.1 |  | 13.9 |  |
| $50,000 or more | 20.7 |  | 20.5 |  | 42.1 |  | 23.0 |  | 18.5 |  | 17.9 |  |
| Missing | 29.6 |  | 32.3 |  | 49.9 |  | 34.7 |  | 25.8 |  | 23.9 |  |
| Employment status |  | 0.6655 |  | 0.0519 |  | * |  | 0.2053 |  | 0.5271 |  | 0.277 |
| Employed / self employed | 23.9 |  | 23.7 |  | 25.1 |  | 26.7 |  | 22.3 |  | 21.4 |  |
| Not employed | 35.4 |  | 35.4 |  | 71.2 |  | 52.0 |  | 35.4 |  | 37.3 |  |
| Retired | 16.8 |  | 12.6 |  | 100.0 |  | 24.2 |  | 12.0 |  | 12.0 |  |
| Homemaker / Student | 29.1 |  | 39.6 |  | 100.0 |  | 32.5 |  | 27.6 |  | 29.7 |  |
| Unable to work | 21.3 |  | 19.3 |  | 100.0 |  | 26.1 |  | 21.3 |  | 19.3 |  |
| Health insurance status |  | 0.9611 |  | 0.4976 |  | 0.9885 |  | 0.2117 |  | 0.6998 |  | 0.8811 |
| Insured | 25.2 |  | 25.1 |  | 47.8 |  | 26.4 |  | 22.8 |  | 23.2 |  |
| Uninsured | 25.4 |  | 28.6 |  | 47.7 |  | 32.9 |  | 24.8 |  | 23.9 |  |
| Geographic residence |  | 0.8078 |  | 0.8635 |  | 0.9568 |  | 0.5112 |  | 0.9568 |  | 0.9021 |
| Urban | 16.3 |  | 18.0 |  | 40.2 |  | 21.3 |  | 14.8 |  | 14.1 |  |
| Rural | 15.2 |  | 18.9 |  | 40.5 |  | 18.0 |  | 12.9 |  | 13.6 |  |

RTRM, Reactions To Race

^1^ Responses weighted according to guidelines provided by the Centers for Disease Control and Prevention

* A p-value was not generated for this portion of the analysis;
